# Tailored, iterative, printed dietary feedback is as effective as group education in improving dietary behaviours: results from a randomised control trial in middle-aged adults with cardiovascular risk factors

**DOI:** 10.1186/1479-5868-8-43

**Published:** 2011-05-20

**Authors:** Janine L Wright, Jillian L Sherriff, Satvinder S Dhaliwal, John CL Mamo

**Affiliations:** 1 School of Public Health, Faculty of Health Science, Curtin University of Technology, Perth, Western Australia, Australia; 2 The Australian Technology Network, Centre for Metabolic Fitness, Perth, Western Australia, Australia; 3 The Curtin Health Innovation Research Institute, Curtin University of Technology, Perth, Western Australia, Australia

## Abstract

**Background:**

Tailored nutrition interventions have been shown to be more effective than non-tailored materials in changing dietary behaviours, particularly fat intake and fruit and vegetable intake. But further research examining efficacy of tailored nutrition education in comparison to other nutrition education methods and across a wider range of dietary behaviours is needed. The Stages to Healthy Eating Patterns Study (STEPs) was an intervention study, in middle-aged adults with cardiovascular risk factors, to examine the effectiveness of printed, tailored, iterative dietary feedback delivered by mail in improving short-term dietary behaviour in the areas of saturated fat, fruit, vegetable and grain and cereal intake.

**Methods:**

STEPs was a 3-month randomised controlled trial with a pre and post-test design. There were three experimental conditions: 1) tailored, iterative, printed dietary feedback (TF) with three instalments mail-delivered over a 3-month period that were re-tailored to most recent assessment of dietary intake, intention to change and assessment of self-adequacy of dietary intake. Tailoring for dietary intake was performed on data from a validated 63-item combination FFQ designed for the purpose 2) small group nutrition education sessions (GE): consisting of two 90-minute dietitian-led small group nutrition education sessions and 3) and a wait-listed control (C) group who completed the dietary measures and socio-demographic questionnaires at baseline and 3-months later. Dietary outcome measures in the areas of saturated fat intake (g), and the intake of fruit (serves), vegetables (serves), grain and cereals as total and wholegrain (serves) were collected using 7-day estimated dietary records. Descriptive statistics, paired t-tests and general linear models adjusted for baseline dietary intake, age and gender were used to examine the effectiveness of different nutrition interventions.

**Results:**

The TF group reported a significantly greater increase in fruit intake (0.3 serves/d P = 0.031) in comparison to GE and the C group. All three intervention groups showed a reduction in total saturated fat intake. GE also had a within-group increase in mean vegetable intake after 3 months, but this increase was not different from changes in the other groups.

**Conclusions:**

In this study, printed, tailored, iterative dietary feedback was more effective than small group nutrition education in improving the short-term fruit intake behaviour, and as effective in improving saturated fat intake of middle-aged adults with cardiovascular risk factors. This showed that a low-level dietary intervention could achieve modest dietary behaviour changes that are of public health significance.

## Background

Cardiovascular disease (CVD) is the leading cause of disease burden in developed nations and by 2010 surpassed infectious disease as the world's leading cause of death and disability [1]. Despite increasing availability of drugs which effectively manage selected risk factors such as hyperlipidemia and hypertension, a population-based prevention approach is widely considered the most effective and affordable strategy to reduce CVD disease prevalence [2]. Approximately 80% of individuals with CVD have modifiable risk factors such as obesity, smoking or poor nutrition [3] and the majority of adults in developed nations such as Australia have one or more modifiable risk factors for CVD [4]. Fortuitously, interventions that produce sustained improvements in personal health practices lead to substantial reductions in the incidence, progression and severity of CVD [3].

Consuming a healthy diet is one of the core set of CVD preventive health strategies known to be effective [2]. What constitutes a healthy diet for both primary and secondary prevention of CVD is also well established, with national and international bodies consistently recommending a diet that is low in saturated and trans-fats and high in fruit, vegetables and grain foods [5,6]. However, in affluent developed countries, few adults eat in a way that consistently meets these recommendations. It has been found that in Australia on average, just 10% of women and 7% of men over the age of 15 years ate five or more serves of vegetables each day and only half of the same people ate two or more serves of fruit per day [7] and in the U.S.A. only about 5% of Americans have dietary patterns that adhere to the vegetable, fruit or dietary fat recommendations of the Healthy People 2010 dietary guidelines [8]. Clearly, there is a need to develop effective behavioural interventions that can positively and cost-effectively change dietary behaviour in adults that have CVD risk factors who are a key target audience for dietary preventive health strategies.

Expert-led, face-to-face education, whether at individual or group level has been the traditional method used for changing health behaviours [9]. Systematic reviews of the literature on interventions to improve eating behaviour in adults found that those which included an interpersonal component such as face-to-face education had consistent and sustained positive effects [9,10]. Aspects of face-to-face education techniques that contribute to their effectiveness in promoting behaviour change have also been identified. For example, nutrition education techniques that individualise the information to the needs of the person show greater effect [9] and within-group education interventions that establish goals appear to be more effective than generic group nutrition counselling [11,12]. However, by their nature, such face-to-face methods are expensive to deliver and are not available to all who can benefit from making dietary changes.

Tailored nutrition education which uses computerised processes to underpin the tailoring and message generation has been suggested as an approach for delivering cost-effective dietary behaviour interventions which capture some of the known benefits of face-to-face education [13,14]. This area of dietary intervention research has developed in response to technological development, greater understanding of the behaviour change process and following demonstrated efficacy in health behaviour change in other areas such as smoking cessation.

Message-tailoring utilizes data on personal attributes to educate individuals to change their behavior [15]. While group-targeted materials are designed to reach particular subgroups of people who share demographic and general health-risk characteristics, tailored messages are individualized based on assessments of personal behaviours, psycho-social characteristics and are tailored to specific targeted outcomes [15,16]. Key features, or points of tailoring used to develop individualised messages with the aim of dietary behavior change have been reviewed [16] and have included: eating behavior feedback [16] and how this compares to peer group norms [16] and dietary goals [17]; psychological characteristics [17] such as the state of the individual's motivation and readiness to change [18]; and the individual's extent of knowledge, perception of the adequacy of own diet [19] and their attitudes towards diet [19]. The duration of a dietary behavior change intervention program using tailored nutrition messages, the optimal number and frequency of messages, as well as the effectiveness of using iterative feedback that builds on previous messages, are also aspects of tailoring design that continue to be investigated [13].

Most randomised trial evidence for the efficacy of tailored nutrition feedback has been in comparison to the provision of generalised nutrition feedback/messages or no feedback at all [13,20]. Many studies have used a tailored message focussed on reducing dietary fat intake [13]. Studies have also reported positive effects of tailored interventions on fruit and vegetable intake, with fewer showing effects on fat intake as well as fruit and vegetable intake [13]. The need to examine the effectiveness of tailored nutrition interventions in comparison to a range of nutrition education methods has been identified [14]. Small group nutrition education is an established and widely used face-to-face behaviour change method [8,9]. However, the effectiveness of tailored nutrition feedback has not been assessed in a randomised controlled trial in comparison to small group nutrition education sessions. Also, despite clear CVD health benefits associated with increased grain and cereal foods consumption [5] no published study has examined the efficacy of tailored feedback in changing this area of eating behaviour, in an intervention that also focuses on saturated fat and fruit and vegetable intake behaviours. Furthermore, the use of iterative feedback as part of the tailoring mechanism across this range of dietary behaviours has not been assessed in a randomised controlled trial.

This study aims to compare the effectiveness, of tailored, iterative, printed dietary feedback, with small group nutrition education sessions and a control group in changing dietary behaviour in the areas of saturated fat, fruit, vegetables and grain and cereal foods. The participants were adults 40 to 65 years eligible for primary or secondary prevention of CVD. We hypothesised that tailored dietary feedback intervention would be more effective than small group nutrition education in changing the targeted dietary behaviours.

## Methods

### Participants

One hundred and seventy eight Australians (85 men and 93 women) were recruited in three phases in August 2002, January 2003 and August 2003. Recruitment was by newspaper advertisement, newspaper community announcements, as well as media publicity on broadcast-radio and community-television. Inclusion criteria were men and women aged between 40 and 65 years requiring primary or secondary prevention of CVD (that is having one or more of the risk factors: overweight or obesity, hypercholesterolemia, hypertension, smoking, family history, or a previous cardiac event). Exclusion criteria were: persons with non-insulin dependent diabetes; non-English speaking; unable to read or write; currently undertaking major dietary modification; major recurrent illness; and not remaining within the vicinity of the study centre for the following 4 months.

Eligibility was assessed by self-report via telephone interview. All participants provided written informed consent and the study was approved by the Curtin University of Technology Human Ethics Committee approval reference number HR20/98

### Study Design

**Figure 1:**
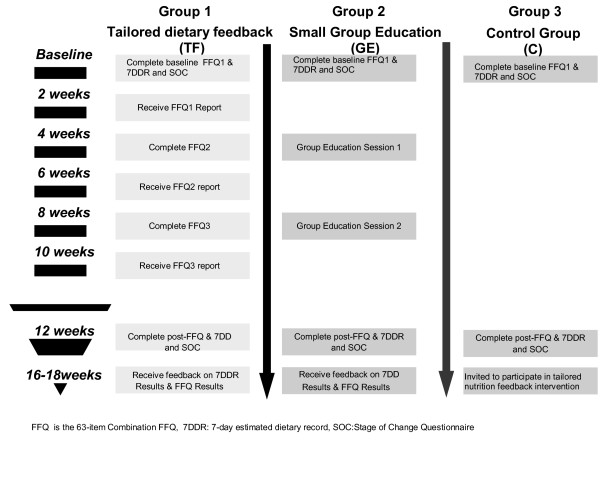
**Study Design of Randomised Controlled Trial**. STEPs - The Stages to Healthy Eating Patterns Study

The Stages to Healthy Eating Patterns Study (STEPs) study, a randomised, controlled trial, was used to determine the effectiveness of two 3-month dietary behaviour change interventions, namely, tailored, iterative, printed dietary feedback delivered via mailed reports (TF) and two 90-minute dietitian-led small group nutrition education sessions (GE). These interventions were compared with each other, and with a control group (C) who were wait-listed for 4-months to receive one of the interventions and who during the intervention timeline completed only baseline and post 3-month socio-demographic questionnaires and dietary measures (Figure 1). The study was conducted from the School of Public Health, Curtin University of Technology with group education sessions conducted in the School's seminar room.

Randomisation schedules were computer-generated for each of the three phases of the RCT using a 3-block design stratified for gender. After completion of baseline measurements, study group allocations were allocated sequentially by research staff not involved in the development of the randomisation schedules. The study was open in design- with participants and researchers aware of their study group allocation.

### Intervention Descriptions

#### Tailored Dietary Feedback (TF) Group

Apart from baseline and study completion measures, participants in the TF group also completed a further two sets of questionnaires, a dietary assessment tool (a combination food frequency questionnaire (FFQ)) and psycho-social questionnaire, at approximately 1-month and 2-months into the intervention (Figure 1). Thus, over the duration of the study participants in the TF intervention group received three, printed tailored dietary feedback reports via the mail prior to their completion of the study outcome measures at 3-months. Each report provided detailed feedback based on the dietary and psycho-social information provided and was received approximately 2-weeks after their completion of the last dietary and psycho-social questionnaires. The dietary feedback reports were the sole form of nutrition education contact that participants in the TF group received.

#### Theoretical Model for TF Group

Overall, the TF intervention was based on elements from the Transtheoretical Model- using both the stages of change and processes of change constructs [21]. The dietary behaviour change sections of the feedback reports were stage tailored and emphasised the key processes of change relevant to that stage [22]. The Precaution Adoption Process model [23]for behaviour change also informed the tailored feedback design. Specifically, in line with this behaviour change theory, misconceptions of dietary adequacy were addressed both in the feedback reports and through the inclusion of the stage classification of pre-contemplative unaware [19].

The tailored feedback reports were generated using a computerised process, with the generated merged word processing document manually formatted for printing purposes. The reports consisted of four parts, with each part addressing one of the four dietary behaviours focused on; saturated fat intake behaviours and the intakes of fruit, vegetables and grain and cereal foods. Each part had two sections. Section 1) gave feedback on both the subject's self-rating of, and their actual, dietary behaviour. Their dietary intake behaviour was compared to the dietary goals, with fruit and vegetable intakes also compared to peer norms. In the iterative (that is the second, third) feedback reports, feedback in this section was also given on changes made between reports and progress. For those identified as being 'precontemplative-unaware' [19] for a particular dietary behaviour (that is, incorrectly perceiving diet behaviour to be adequate and therefore with no intention to change), there was additional tailoring identifying how belief in the adequacy of their dietary intake pattern did not match the information analysed from the FFQ. Section 2) of the feedback report provided behaviour change content relevant to the person's stage of change for that food behaviour, as identified by key processes of change for that stage [22]. For example, those 'pre-contemplative' for a dietary behaviour had information and activities that encouraged the consideration of the benefits of change and weighing the pros and cons of change. As another example, someone identified as being in 'action' for a dietary behaviour was given positive reinforcement for 'doing it', and were asked to: remember the motivations behind the action; set a specific goal; set up reward and support system and to plan for difficult situations.

#### Small Group Nutrition Education (GE) Group

The small group nutrition education program consisted of two 90-minute dietitian-led education sessions, designed for a group size of 10 to 15 persons. Each session focused on the same four dietary messages, namely, (reducing) saturated fat related intake and behaviours and (increasing) intake of fruit vegetables and cereal and grain food (in particular wholegrains). The content and activities of the group education sessions, such as label-reading activities and goal setting activities, were designed in line with current dietitian-run nutrition education programs for persons at risk of CVD, or receiving information for secondary prevention of CVD. To minimise differences between the group education sessions, all small group nutrition education sessions, throughout all three phases of the RCT, were conducted by the same dietitian.

#### Control (C) Group

The control group completed all baseline questionnaires and completed all dietary measures (as for the TF and GE groups), at baseline and then 3-months later. The participants were then invited to receive the TF intervention (for phase 1 and 2 of the RCT) and the GE intervention (for phase 3 of the RCT).

### Measurements

Baseline measurements (socio-demographic and psychosocial questions and dietary measures) occurred before randomisation with outcome measures assessed at study completion (3-months post-baseline). Basic socio-demographic data was collected and psychosocial questions assessing stage of change, self-efficacy measures, perception of dietary barriers and knowledge related to fruit, vegetable, grain and cereal foods intake and saturated fat intake behaviours were also assessed at baseline and study completion. All questionnaires were self-administered.

#### Psycho-social instruments

**Figure 2:**
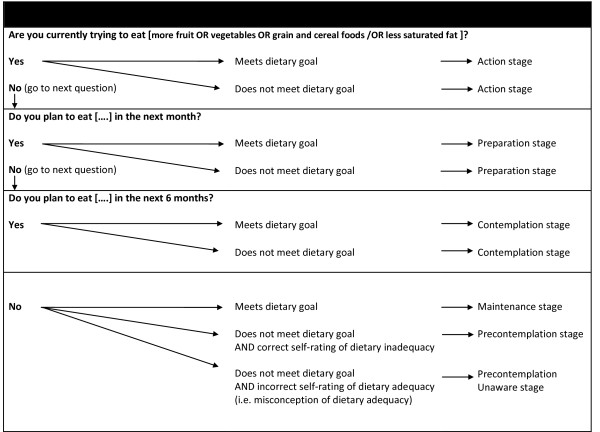
**Stages of Change questionnaire: question structure and stage classification details**. Stage of change classification included both behavioural intention and objective assessment of dietary intake.

Stage of change (SOC) questions for fruit, vegetables, grain and cereal foods and saturated fat intake behaviours were based on those developed and validated by Lechner et al. [19] and Ling and Horwath [24]. Sample question structure and staging algorithm for SOC is detailed in Figure 2. Stage classification incorporated objective assessment of food behaviour by the combination FFQ. Self efficacy regarding ability to: increase the intake of fruit, vegetable and, grain and cereal foods; choose low fat dairy products; choose trimmed and lean meat and poultry; reduce consumption of high fat sweets and desserts; reduce consumption of high fat snacks and take away foods; and reduce or substitute use of high saturated fat in cooking or added to foods was assessed with a one-item question, using a 4-point scale (1-very confident, 4- not at all confident) as used by Brug et al. [17] and Campbell et al [25]. Perceptions of the benefits and barriers to change for each food behaviour area, and knowledge of current dietary goals were assessed using the standardised questions assessing these areas included in the Western Australian Health Department Nutrition Monitoring Survey [26] and the Australian 1995 National Nutrition Survey [27].

#### Seven-day estimated dietary records

Baseline dietary intake, and dietary intake at study completion were assessed using a self-administered 7-day estimated food record booklet which included food photographs. These food record booklets were modified, with permission, to suit an Australian setting from those used and validated by Raats and Geekie [28]. The booklet consisted of two pages of instructions, a sample day record, 12 pages for recording food and drinks and six pages of photographs depicting reference 'medium' portion sizes of foods. The booklet also included two fold-out flaps with descriptions of 'medium' portion sizes. Participants were asked to describe 'amount' eaten in terms of photographs and list provided, in terms of household measures and in terms or weights taken from food packaging. Participants received a detailed 20-minute telephone-based training session on the use of the food records which included a number of sample recording scenarios to explain how to best complete the food records.

#### Combination Food Frequency Questionnaire - the tailoring dietary assessment tool

**Figure 3:**
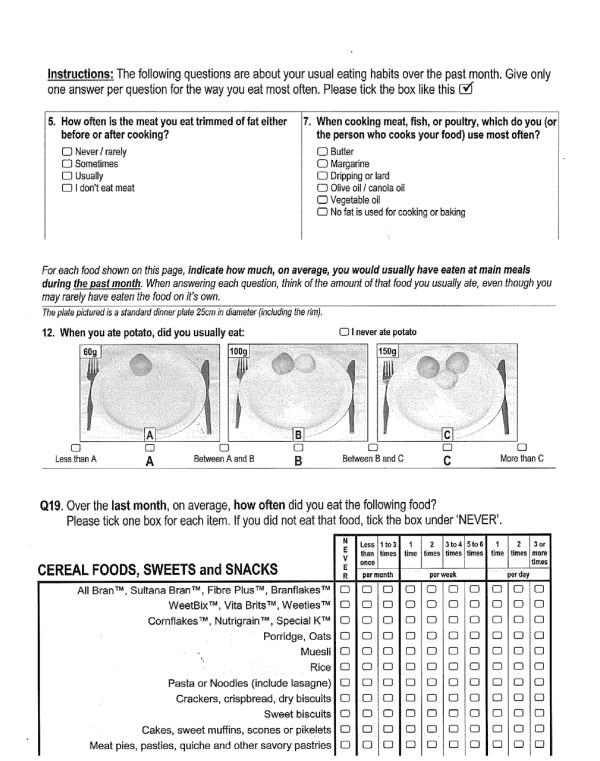
**Question examples from the combination food frequency questionnaire used as the tailoring dietary assessment tool**. The combination FFQ included a combination of questions types. Shown are examples of qualitative questions on food choice preferences, semi- quantitative items which required participant definition of serve size, and frequency only questions

All participants completed a 63-item validated [29,30] combination FFQ at baseline and study completion. The FFQ included a combination of questions types: quantitative items which required the participant definition of serve size, frequency only questions and qualitative questions on food choice preferences (eg type of milk and fat spread use). Figure 3 provides examples of the FFQ format.

Participants in the TF group also completed the FFQ at 1-month and 2- months into the study, as the FFQ was the dietary assessment tool used as the basis for providing the detailed dietary feedback. This questionnaire, based in several sections on the 73-item 1998/99 version of the Anti-Cancer Council of Victoria Food Frequency Questionnaire [31], was designed and its measurement characteristics tested specifically for the process of providing tailored dietary feedback in this trial. The FFQ assessed the previous months' dietary behaviours allowing calculation of daily average intake in serves (as defined by the Australian food selection guide: The Australian Guide to Healthy Eating ) [32] for fruit, vegetables and grain and cereal foods. These serve intakes were then able to be compared to current food-based recommendations, specifically, 2 or more serves of fruit per day, 5 or more serves of vegetables [33] and for women 4 serves of grain and cereal foods and for men 5 serves of grain and cereal foods [32]. The FFQ analysis also allowed determination of whether saturated fat intake behaviours met the dietary goal for those with a risk factor for CVD [34].

### Data Analysis

#### Dietary Analysis

Nutrient intake from the 7-day estimated food record completed at baseline and post-intervention were determined using FoodWorks Version 3.02 (Xyris Software, Brisbane, Queensland, Australia) utilising the AUSNUT 1999 database (Food Standards Australia). Nutrient outcome variables were daily averages of saturated fat (g/d), and fibre intake (g/d) and saturated fat intake as percentage of total dietary energy intake. The dataset of analysed nutrient data was for 139 participants: n = 48 for TF group, n = 41 for GE group and n = 50 for C group.

Study randomisation followed complete collection of all baseline data, and from this point onwards no further participants were excluded from the study. Due to corruption of some 7-day estimated dietary record nutrient data the analysed dataset differed from the point of randomisation dataset by n = 39 (n = 10 TF, n = 17 GE and n = 12 C group).

Food group data (fruit, fruit juice, vegetables (with potato classified separately), grain and cereal foods, and wholegrains) was determined through export of FoodWorks diet record food lists into Microsoft Access 2000. All foods and drinks eaten by participants in the 7-day estimated dietary record were coded into food groups according to the specifications of the Australian Guide to Healthy Eating [32] and then re-linked to each participants dietary record to allow calculation of 7-day food group totals and daily averages. Food group outcome variables were: average daily serves of fruit including fruit juice (to a maximum of an average of 1 serve/d), serves of vegetables with and without potato (non-fried), total serves of grain and cereal foods and serves of wholegrains only. The dataset of analysed food group data was for 153 participants: n = 48 for TF group, n = 47 for GE group and n = 58 for C group. Due to corruption of some 7-day estimated dietary record data the analysed dataset for food group data differed from the point of randomisation dataset by n = 25 (n = 10 TF, n = 11 GE and n = 4 C group).

The combination FFQ was analysed using a developed Microsoft Excel 2000 algorithm which generated data on daily average serves of: fruits (including a maximum of 1 serve per day from fruit juice), vegetables (including non-fried potatoes), and cereal and grain foods. Saturated fat intake (g) was then compared to a cut-off for males and females determined through a receiver operator curve (ROC) validation process [29] on whether they met the dietary goal for saturated fat.

#### Statistical Analysis

All data were analysed using the Statistical Package for the Social Sciences (SPSS Version 17.0, Chicago, IL, USA). Intention-to-treat analysis was performed with all participants included in the analysis according to original group allocation and follow up was maximised regardless of attendance in the case of the GE group. The LOCF (Last Observation Carried Forward) was used as a means for imputing missing 3-month follow up values [35] for those subjects that provided baseline data.

Log transformation was used, as needed, for variables that were not normally distributed. Treatment effects were examined using general linear models (ANCOVA) adjusted for sex, age and baseline values with a priori contrasts comparing each treatment group with the control group. Categorical data were compared using chi-squared tests. P < 0.05 was considered significant.

## Results

Results are presented based on an intention to treat analyses, using the LOCF (Last Observation Carried Forward) as a means for imputing missing 3-month follow up values [35] for those subjects that provided baseline data.

Table 1 describes the demographic characteristics of the participants at randomisation. There were no differences between study groups at baseline for any demographic characteristics (data not shown). Comparison between characteristics of those within the dataset of analysed nutrient and the dataset of analysed food group data at baseline (Table 1) and all those randomised, found no differences in demographic characteristics (data not shown).

**Table 1:** Baseline characteristics of subjects at randomisation according to intervention groups: n = 178

	Tailored dietary feedback group		Small Group nutrition education group		Control Group	
	*n*		*n*		*n*	
**Gender (M/F)**	58	29/29	58	26/32	62	30/32
**Education, y**	***n***	%	***n***	%	***n***	%
**12 or less (%)**	33	57	31	53	28	45
**>12 (%)**	25	43	27	47	34	55
**Smokers (%)**	0	0	1	2	3	5
**Body Mass Index categories (%)**	***n***	%	***n***	%	***n***	%
**up to 25**	11	20	11	20	15	25
**>25 to 30**	20	34	24	43	23	38
**>30 to 35**	20	34	11	20	15	25
**>35 to 40**	1	2	5	9	5	8
**>40**	2	3	5	9	3	5
	***n***	**mean (SD)**	***n***	**mean (SD)**	***n***	**mean (SD)**
**Age (y)**	56	54.6 (7.0)	58	53.4 (6.5)	62	54.0 (7.0)
**Weight (kg)**	56	82.8 (14.5)	58	83.9 (18.7)	61	85.3 (21.0)
**Body Mass Index **	54	29.0 (4.6)	56	30.1 (6.1)	61	29.0 (5.7)

Baseline nutrient intake data did not differ between study groups within the dataset of analysed nutrient data. For analysed food group data, the TF group's mean baseline intake of total vegetables not including potatoes at 3.0 serves/d (SEM 0.2) (Table 2) was higher than the GE group (p = 0.024) and the C group (p = 0.025). The mean total vegetable intake including potatoes of the TF at 3.4 serves/d (SEM 0.2) was also greater (p = 0.06) than that of GE baseline intake at 2.7 serves/d (SEM 0.2) and the control group (p = 0.015) intake of 2.8 serves/d (SEM 0.2).

**Table 2:** Dietary behaviour changes pre- and post 3-months: comparison between and within intervention groups- intention-to-treat analysis

		Baseline	Follow-up	Difference	Comparison of group change to Control change^a^
					p-value^b^
**Saturated Fat (g/day)**	TF	26.5 (1.2)	24.1 (1.5)	-2.4**	0.561
	GE	26.6 (1.7)	22.7 (1.1)	-4.9**	0.077
	C	27.3 (1.1)	25.0 (1.2)	-2.3**	...

**Fruit (serves/d)**	TF	1.8 (0.2)	2.1 (0.1)	+0.3	0.047
	GE	2.0 (0.1)	1.7 (0.2)	-0.2	0.780
	C	1.7 (0.1)	1.7 (0.1)	0	...

**Vegetables without potatoes (serves/d)**	TF	3.0 (0.2)	2.9 (0.2)	-0.1	0.685
	GE	2.4 (0.1)	2.9 (0.2)	+0.5**	0.108
	C	2.4 (0.2)	2.5 (0.2)	+0.1	...

**Grains-total (serves/d)**	TF	2.4 (0.2)	2.3 (0.2)	-0.1	0.359
	GE	2.5 (0.2)	2.5 (0.1)	0.0	0.690
	C	2.5 (0.1)	2.5 (0.1)	0.0	...

**Wholegrains (serves/d)**	TF	1.2(0.1)	1.3 (0.1)	+0.1	0.094
	G	1.1 (0.1)	1.2 (0.1)	+0.1	0.155
	C	1.2 (0.1)	1.0 (0.1)	-0.2	...

Tailored Dietary Feedback (TF); Small Group Nutrition Education (GE) and Control Group (C) ^a ^Study group effects were examined using general linear models adjusted for gender, age and baseline dietary values ^b^*p *values for dietary differences are based on t-tests after general linear models (ANCOVA), the a priori contrasts compared each intervention group with the control group **P < 0.05 based on paired t-test within study group results

### Dietary Changes

Table 2 presents the comparison across the study groups for daily consumption of the dietary components focused on in the interventions, that is saturated fat, and fruit, vegetable and cereal and grains intake at baseline, and post-intervention at 3-months. Between-group comparisons found that one area of dietary behaviour change was different amongst the study groups. The TF group was found to be more effective than GE and C group in increasing fruit intake. Contrasts between the groups found that the TF group increase in fruit intake of 0.3 serves/d or a 16.7% increase from baseline, was significantly different for both the TF-GE comparison (p = 0.031) in whom mean fruit intake reduced by 0.2 serves/d and TF-C group comparison (p = 0.047) where fruit intake was unchanged during the intervention period.

Within-study group comparisons found that all three study groups were effective in significantly reducing their total intake of saturated fat (g/d) (Table 2). Comparison of the 3-month and baseline results revealed that participants in the TF group decreased their intake of total saturated fat by 9.1%, participants in the GE group decreased their saturated fat intake by 18.4% and those in the C group by 8.4%. Examination of between-group intervention effects found no difference in the ability of the TF to reduce saturated fat intake compared with the GE and the C groups.

There were no between-study group effects for vegetable intake (both with and without non-fried potatoes) or for measures of cereal and grain intake (both total and wholegrain alone). Within the GE group the mean intake of vegetables was significantly increased over the 3-months with an increase of 0.5 serves/d (20.8% increase on baseline), but this difference was not significant when compared to the changes of the TF and C groups.

The dietary behaviour showing the least impact from either of the dietary interventions or control group was cereal and grains intake. Within-study group or between-study group differences found for grain and cereal intake were not evident, either as total serves, or wholegrain serves only.

## Discussion

Overall, this randomised controlled study demonstrated that tailored, iterative, printed dietary feedback was more effective than small group nutrition education in increasing fruit intake over a 3-month period in adults at risk of CVD, and as effective as group education in reducing total saturated fat intake. The other areas of dietary behaviour focus in this study, namely intake of vegetables and cereals and grains, were not additionally changed by either dietary intervention, in comparison to the no-education control group who completed the baseline and 3-month post-test dietary measures only.

The increase in fruit intake of 16.7% seen in the TF group meant their mean intake of fruit at 3 months post-baseline was 2.1 serves/day and met the current Australian dietary recommendations for fruit of at least 2 serves/day [32,33]. Furthermore, the greater effectiveness of the TF intervention was combined with a reduction in total saturated fat intake which was not found to be significantly different from that gained from the more resource-intensive GE intervention.

Such dietary improvements in fruit intake and saturated fat achieved from the TF intervention are meaningful in public health terms [33]. These modest dietary changes, when reached by the population at large have been shown to be of substantial health benefit [33].

The findings of this study show areas of agreement with the mixed results published on the effectiveness of tailored, nutrition feedback. Other studies have found tailored feedback to be effective in reducing saturated fat intake, and able to effect both a dietary fat behaviour and intake of fruit and/or vegetable [13]. This study's results are also consistent with a number of studies showing mixed effects in terms of the efficacy of a tailored intervention in improving multiple dietary behaviours [13,20]. The inclusion of grain and cereal behaviours as a dietary target for a tailored dietary behaviour change intervention, in addition to saturated fat, fruit and vegetable intake behaviours has not been previously investigated. In terms of the between dietary intervention comparisons it is difficult to compare this study's results to those of others, as the comparison of tailored dietary feedback to small group nutrition education was in this study led by a dietitian.

Of all the dietary behaviours at baseline- mean fruit intake was closest to the dietary goal and thus on average, the fruit section of the tailored feedback reports would have identified the smallest dietary behaviour change required to meet the goal. It has been hypothesised that strength of tailored feedback is in providing objective individualised dietary feedback which provides a more accurate assessment of where a person's dietary intake is in comparison to goal and in this way tailored feedback incorporates an aspect of interpersonal dietary counselling that may not be part of small group nutrition education [19,36]. It may be that when this objective feedback identifies that the dietary changes recommended are small, change processes supported by the non-face-to-face TF intervention shows effectiveness. The cognitive factors underpinning the processes of dietary changes will be a topic of further analysis and publication.

This dietary improvement in fruit intake by the TF group was achieved from a baseline mean intake of 1.7 serves per day which is within the population estimate of mean intake fruit intake of West Australian adults at that time, at 1.8 serves/day (95% CI 1.7-1.9) [33]. It should also be noted that the improvement was made in the midst of extensive social marketing campaign for the increase of fruit and vegetable intake, the Go for 2 and 5 ™ campaign [33].

In this study small group nutrition education was found to be no more effective in producing dietary behaviour change than either tailored feedback or a control group. The reduction in total intake of saturated fat of 18.4% for the GE group between baseline and post-test was significant as a change within that group and approached significance (p = 0.077) in the between-group study effect. The GE group also demonstrated dietary behaviour improvements from baseline to post-test 3 months later in vegetable intake. Thus, the study results were consistent with the evidence base that GE is a successful dietary behaviour change technique at least for some dietary behaviours.

The positive impact of completing detailed dietary measures without further input on dietary behaviours, as seen in this study in the reduction of saturated fat in the C group, has been shown in previous research [37]. The act of completing 7-day estimated food records and dietary questionnaires is an intensive act of dietary behaviour focus and is a form of dietary self-monitoring that can be considered low-level dietary change intervention in itself.

No dietary improvements were seen in the area of grain and cereal intakes for any of the study groups. Other RCT to date have not examined the ability of TF to alter grain and cereal intake (with a focus on wholegrain intake). The inclusion of grains and cereals in the nutrition education interventions (TF and GE) was seen to be important in providing a more complete dietary behaviour change message linked to the reduction of risk of CVD as there is increasing evidence that the increased consumption of grain and cereals, particularly wholegrain, reduces CVD risk [5]. However, the public health nutrition messages on grain and cereal foods, with their provision of greater amounts of dietary energy, and messages about the role of carbohydrates in weight control, are more complex than consistently positive messages in regards to fruit and vegetable intake. Grain and cereal foods have also not had the same focus in health promotion social marketing that the increase in fruit and vegetable intake, and decreasing of fat intake (particularly saturated fat) has.

In this study recruitment was through advertisement and thus study participants would show volunteer bias and have an increased interest in dietary change. This limits the generalisability of the study's results. However, this volunteer group may represent well a group that accesses a tailored dietary feedback intervention, as applications of this intervention could be provided across the internet to person's with CVD risk who 'volunteer'. The participants were found to be representative of the demographic characteristics and dietary characteristics of Australian adults at CVD risk in the 40-65 year old age range. Also, this study with its assessment of change over 3-months looks at only short-term dietary behaviour change. As such it is not known whether the changes from the interventions would have been sustained. These questions would be answered by further research of longer duration.

This study focused on the efficacy of TF to promote dietary behaviour change in comparison to the more expensive face-to-face dietary intervention of GE, and a wait-listed C group. As such it was dietary behaviours that were the focus and the outcome measures for the study. The form of dietary measure used, the 7-day estimated record, is considered a robust tool for determining the food group based and saturated fat intake related dietary outcome measures used in this study [38]. Furthermore, that this study included this measure as an independent measure of dietary change is an improvement on most previous studies. In most studies assessing the effectiveness of tailored feedback, dietary questionnaires only have been used to measure dietary change and often these questionnaires have been the intervention tool itself [13,14].

This study with its comparison of tailored, iterative, printed dietary feedback to the commonly used nutrition education technique of group education sessions is the first of its kind. It has added to the knowledge base on the effectiveness of an intervention that does not include face-to-face education can have on dietary behaviour. This trial provides evidence that over the short-term that tailored, iterative feedback was more effective than group education in improving fruit intake, and was as effective in reducing total saturated fat intake in adults 40-65 years at risk of CVD. Neither tailored feedback nor group education demonstrated greater efficacy in changing the intake of vegetables, or grain and cereals in this subject group.

## Conclusions

In this study, tailored, iterative, printed dietary feedback was more effective than small group nutrition education in improving the short-term fruit intake behaviour, and as effective in improving saturated fat intake of middle-aged adults with cardiovascular risk factors. This showed that a low-level dietary intervention, which did not include face-to face contact, could achieve modest dietary behaviour changes that are of public health significance.

## List of Abbreviations

C: control; CI: confidence interval; CVD: cardiovascular disease; FFQ: food frequency questionnaire; GE: group education; RCT: randomised controlled trial SEM-standard error of the mean; STEPs: The Stages to Healthy Eating Patterns Study; TF: tailored feedback.

## Competing interests

The authors declare that they have no competing interests.

## Authors' contributions

JW conceived and designed the study, collected, analysed and interpreted the data, and led in the writing of the manuscript. JS participated in the design of the RCT, analysis and interpretation of data and helped to draft the manuscript. SD participated in the analysis and interpretation of data and helped to draft the manuscript. JM participated in the analysis and interpretation of data, reviewed and edited the manuscript. All authors read and approved the final manuscript.
